# Socioeconomic disparities in in-hospital mortality and 30-day hospital readmission rates among people with eating disorders: a retrospective cohort study

**DOI:** 10.1186/s40337-025-01422-8

**Published:** 2025-10-29

**Authors:** Sakiko Ohashi, S. Bryn Austin, Tracy Richmond, Ichiro Kawachi

**Affiliations:** 1 Okayama University Medical School, 2-5-1 Shikata-cho, Kita-ku, Okayama, 700-8558 Japan; 2 Harvard T.H. Chan School of Public Health, 677 Huntington Ave, Boston, MA 02115 USA; 3 Boston Children’s Hospital, 300 Longwood Avenue, Boston, MA 02115 USA

**Keywords:** Eating disorders, Socioeconomic disparities, Readmissions, In-hospital mortality, Socioeconomic status, Income, Insurance status, Medicare, Medicaid, Nationwide readmissions database

## Abstract

**Background:**

We sought to examine socioeconomic disparities in 30-day hospital readmissions and in-hospital mortality among admissions of people with eating disorders in a U.S. national sample.

**Methods:**

Using the 2019 Healthcare Cost and Utilization Project Nationwide Readmissions Database (HCUP-NRD), we analyzed data from 13,986 admissions with a primary or secondary diagnosis of eating disorders. Multivariable logistic regression assessed associations between socioeconomic status (SES), insurance status, and 30-day readmission, as well as in-hospital mortality. SES was categorized into quartiles based on neighborhood median income from patients’ postal codes. Insurance status included Medicare, Medicaid, private insurance, and other. Generalized Estimating Equations (GEE) accounted for facility clustering.

**Results:**

Of 13,986 admissions, 693 were readmissions. Admissions of people with eating disorders in the highest income quartile had significantly higher odds of readmission (OR = 1.60, 95% CI = 1.23–2.10) compared to those in the lowest quartile. Admissions covered by Medicaid (OR = 0.71, 95% CI = 0.57–0.89) or private insurance (OR = 0.66, 95% CI = 0.53–0.83) had lower odds of readmission compared to admissions covered by Medicare. No significant differences in readmission rates were observed across hospitals located in different geographic areas nor across hospitals with differing ownership. In-hospital mortality was highest among admissions of people with eating disorders insured by Medicare (1.35%) and lowest among those with private insurance (0.39%) (OR = 0.48, 95% CI = 0.27–0.84).

**Conclusion:**

People with eating disorders from higher SES backgrounds had higher readmission rates but lower in-hospital mortality, potentially indicating that these people may be receiving a more intensive level of outpatient care. Higher readmission rates may paradoxically indicate continued engagement in follow-up care. However, this interpretation remains speculative and further research is needed to explore the mechanisms behind these disparities, particularly focusing on access to care for people with eating disorders from lower-income backgrounds.

## Plain language summary

We sought to examine how income and insurance status affect hospital outcomes for people with eating disorders in a 2019 U.S. national sample, focusing on two main outcomes—whether readmission to a hospital occurred within 30 days, and whether an admission ended in death during hospital stay. We found that admissions of people with eating disorders living in higher-income areas were more likely to be readmitted. Separately, we found that admissions of those with Medicaid or private insurance were less likely to be readmitted compared to those with Medicare. Lastly, we found that those with private insurance were less likely to die during their hospital stay compared to those with Medicare. These findings, although speculative, may suggest that people with eating disorders from higher socioeconomic backgrounds may have better access to follow-up care. Further research is needed to understand why these disparities exist, especially focusing on whether or not people with eating disorders from lower socioeconomic backgrounds are able to access the care they need.

## Background

With an estimated lifetime prevalence of 8.6% among females and 4.1% among males, [1] eating disorders have among the highest case fatality rates of all mental disorders [2]. A meta-review conducted by Chesney et al. on the risks of all-cause and suicide mortality in mental disorders found anorexia nervosa to have the second highest all-cause standardized mortality ratio (5.9) among psychiatric disorders, following substance use disorders [3]. Bulimia nervosa and eating disorders not otherwise specified also demonstrated elevated standardized mortality ratios (both 1.9) [3]. Duration until treatment starts varies by disorder: anorexia nervosa has an average duration of approximately 2.5 years, while binge eating disorders can last nearly 6 years [4]. Typically characterized by a high risk of relapse after stabilization, [5] many individuals need multiple engagements in higher levels of care throughout their treatment journey [6]. 

Treatment for eating disorders include, in increasing order of severity: outpatient care, intensive outpatient programs, partial hospitalizations, residential treatment, and inpatient hospitalizations, with inpatient hospitalizations being reserved for the most severely impacted individuals [6]. Following inpatient care, patients often transition to lower levels of care [6]. Each level of care addresses specific needs, and treatment failure at any stage typically results in recommendations for an increased level of care [6]. 

The most recent systematic review of the literature found no consistent evidence to support a socioeconomic gradient in the incidence of eating disorders [7]. Earlier reports of a higher incidence of eating disorders among high SES groups may partly reflect an artifact of differential rates of diagnosis and treatment [8]. Individuals from less affluent backgrounds are less likely to receive proper screening and treatment, due to lack of access to mental health services [9]. Even when these individuals do seek care, clinicians may be less inclined to ask about and accurately diagnose eating disorder symptoms among those who do not conform to the affluence stereotype, thereby further perpetuating the misconception that eating disorders are a disease of the affluent [9–11]. 

Several studies suggest an association between SES and all-cause hospital readmission, whereby higher readmission rates are observed among people from lower income groups [12–15]. One contributing factor is the limited access to primary or post-discharge care services experienced by low SES individuals, due to issues of affordability and availability [16, 17]. Readmissions in people with eating disorders are relatively common, with one study finding approximately 36% requiring readmission within a year of discharge, often reflecting the complexities of managing these conditions [5, 6]. Factors contributing to readmissions may include the chronic nature of eating disorders, insufficient follow-up care, and challenges in transitioning to outpatient programs or other lower levels of care [6]. Similar patterns are observed in other mental health conditions, where readmissions can signify inadequate continuity of care or lack of engagement with treatment options [18, 19]. 

Given the complexity of eating disorders and their treatment, people hospitalized for these conditions typically do not leave inpatient hospitalization ‘cured’ or ‘recovered’ but rather stabilized, with an ongoing risk for worsening symptoms. In this context, more outpatient care may actually lead to faster recognition of clinical deterioration and thus hospital readmissions, and may indicate that people with eating disorders are remaining engaged in necessary care. For instance, a study by Di Vitantonio et al. found that people with eating disorders who experienced better transitional and continuity of care during their inpatient or residential treatment were more likely to be readmitted [20]. The authors suggested that these findings indicate individuals with positive treatment experiences may be more inclined to seek help or be encouraged to return for care if they experience a relapse in symptoms [20]. Within this framework, hospital readmissions can be interpreted as a sign of engagement in necessary care, rather than simply reflecting treatment failures.

Despite the frequency of readmissions and the complexities involved in their interpretation, there remains a gap in understanding the specific predictors of readmission among people with eating disorders. One of the limited studies available, conducted in Australia, examined short term 28-day hospital readmission in children and adolescents with anorexia nervosa and found that male gender, low socioeconomic status, the presence of certain co-occurring illnesses, and residence in remote locations all increased the likelihood of readmission [21]. The authors highlighted the lack of access to specialist mental health services in rural regions of Australia as a potential explanation behind the trend [21]. Other studies, such as those by Marzola et al. and Di Vitantonio et al., [5, 20] have explored predictors of readmission among people with eating disorders, but their focus has largely been on clinical factors. Marzola et al., for example, found that a lower body mass index and longer illness duration predicted shorter time to readmission in an Italian cohort of individuals with anorexia nervosa [5]. Di Vitantonio et al., using a U.S. sample, identified factors such as transitional care quality and nasogastric feeding as predictors of readmission [20]. However, neither study examined the role of socioeconomic status or insurance coverage in shaping these outcomes. To our knowledge, no large-scale U.S.-based studies have comprehensively evaluated how social and economic factors influence readmission among people with eating disorders.

Similarly, there is limited research on how social determinants may influence mortality in people with eating disorders. Although direct studies linking socioeconomic status to mortality in people with eating disorders are scarce, disparities in access to care—particularly in early intervention and continuity of treatment—may have downstream effects on mortality, with untreated or undertreated eating disorders having greater medical severity and an increased risk of premature death.

In the present study, we sought to examine whether there are socioeconomic disparities in short term 30-day hospital readmission and in-hospital mortality for people with eating disorders in a U.S. cohort. Our hypothesis was that individuals of lower SES diagnosed with eating disorders, as well as those from rural locations, would be readmitted to hospitals at a higher rate than individuals of higher SES or non-rural individuals, respectively. We also hypothesized that lower SES individuals would exhibit higher odds of in-hospital mortality compared to those of higher SES.

## Methods

We conducted a cross-sectional analysis using the U.S. Healthcare Cost and Utilization Project Nationwide Readmissions Database (HCUP-NRD) from 2019. The HCUP-NRD provides national estimates of eating disorder-related hospital readmissions for children, adolescents, and adults. The NRD is the largest publicly available all-cause hospital readmission database in the United States and contains data from over 18 million discharges annually, which represents over 60% of all U.S. hospitalizations [22]. Weights are applied to each discharge to produce national estimates, amounting to roughly 35 million weighted discharges annually [22]. While more recent data (up to 2022) are available, we selected the 2019 dataset to avoid the confounding impact of the COVID-19 pandemic on hospitalization patterns and outcomes. Our aim was to ensure that the findings reflect typical pre-pandemic healthcare delivery, unaffected by the substantial disruptions and changes in clinical practice that occurred during the pandemic.

The study sample consisted of all hospital admissions (including but not limited to psychiatric inpatient stays) of individuals between 8 and 64 years of age in the HCUP-NRD from 2019 with either a principal or secondary diagnosis of eating disorders, identified using the International Classification of Diseases, Tenth Revision, Clinical Modification (ICD-10-CM) code F50. This code encompasses various eating disorders, specifically including anorexia nervosa, bulimia nervosa, binge eating disorder, other specified eating disorders, and unspecified eating disorders.

Diagnoses related to other medical conditions were excluded as outlined below. Admissions were excluded from the analysis if they had missing lengths of stay, or were discharged against medical advice. Admissions were also excluded if individuals were residents of a state different from the one where the hospital was located, as patients are identified by State-specific linkage numbers and therefore cannot be tracked across different states. Moreover, as the patient-specific identifiers do not carry over across data years, admissions during the last 30 days of 2019 were excluded in order to accurately capture 30-day readmissions. In order to filter out diagnoses of disordered eating unrelated to body image issues, we excluded admissions of people diagnosed with avoidant/ restrictive food intake disorder (ARFID), characterized by disordered eating behaviors without body image disturbances, [23] and admissions under 8 years of age. This decision was made based on findings that cases of eating disorders with acknowledged body image disturbances are exceedingly rare in those younger than 8 years [24]. Admissions of individuals 65 years of age and older were also excluded, as the majority had a diagnosis of other specified eating disorder. This exclusion was based on concerns that this diagnosis might reflect a different underlying process than disordered eating related to body image issues. IRB approval was not sought as the data in the HCUP-NRD is fully de-identified and does not involve human subjects according to federal regulations.

Our main exposures of interest were socioeconomic status (estimated using neighborhood median income), insurance status, and hospital location. It should be noted that while income is a common proxy for SES in administrative data, it captures only one aspect of SES and does not account for other relevant factors such as education, occupation, or access to social resources. Neighborhood median incomes were categorized into quartiles estimated based on the median household income of residents in the individual’s postal code ($1–47,999, $48,000–60,999, $61,000 - $81,999, and $82,000+) [22]. Insurance status included Medicare (federal health insurance for individuals aged 65 and older or those with certain disabilities), Medicaid (a joint federal and state program that helps cover medical costs for individuals with limited income), private insurance (insurance offered by private companies), and self-pay/no charge/other. Geographic location was stratified into three levels: large metropolitan areas with at least 1 million residents, small metropolitan areas with less than 1 million residents, and micropolitan areas with at least 10,000 but less than 50,000 residents and a non-urban residual, adapted from the Urban Influence Codes [22]. 

Covariates included age group (children and adolescents aged 8–17, young adults aged 18–30, midlife aged 31–55, or older adults 56–64 years), sex (male or female), length of stay (< 4, 4–7, or >7 days), disposition at discharge (routine discharge, transferred to a short-term hospital, transferred to another type of facility such as skilled nursing facilities or intermediate care facilities, or discharged to home health care), the number of co-occurring physical and mental diagnoses (0, 1–3, 4–6, or 7+) and the specific co-occurring diagnoses present out of the 10 most frequently observed co-occurring diagnoses overall (anxiety, depressive episode, fluid, electrolyte, and acid-base balance disorders, reaction to severe stress (such as post-traumatic stress disorder) and adjustment disorders, malnutrition, nicotine dependence, gastro-esophageal reflux disease, bipolar disorder, hypertensive diseases, and volume depletion). Hospital ownership (public, private non-profit, or private for-profit) was also included as several studies have found public hospitals to have higher psychiatric readmission rates than both private non-profit and for-profit hospitals [25, 26]. 

The first outcome in this study was all-cause 30-day readmission, as monitoring 30-day follow-up after hospitalization discharge is a widely used healthcare effectiveness metric from the National Committee on Quality Assurance’s Health Plan Employer Data and Information Set (HEDIS FUH) [27]. This approach aligns with standard practices in health services research, where the 30-day readmission rate is a widely used quality indicator, facilitating comparability across studies and healthcare settings. Moreover, the Centers for Medicare & Medicaid Services have incorporated 30-day all-cause unplanned readmission rates into the Inpatient Psychiatric Facility Quality Reporting program, further highlighting its significance as a key performance metric in mental health care [28]. The readmission variables were defined as any individual readmitted within 30 days of discharge from their index hospitalization. Consistent with HCUPnet’s methods for calculating readmissions, any subsequent hospitalizations after the first readmission within the 30-day period were not counted as a 30-day readmission [29]. Hospitalizations occurring after this initial 30-day period post index hospitalization were treated as a new index hospitalization.

The second outcome in this study was the number of total admissions ending in death before discharge between the different income quartiles and insurance statuses. In-hospital mortality was defined as admissions where the database indicated that the individual died during hospitalization. Deaths occurring after discharge were not included as they were not captured in the database.

Statistical analysis was conducted with STATA Version 18 (StataCorp, College Station, TX). Thirty-day readmission rates were calculated using the number of admissions discharged alive after their index admission as the denominator. Multivariable logistic regression was used to test for associations between various individual and hospital characteristics and risk of readmission, with the unit of analysis being admissions. All covariates were entered simultaneously into the model (ENTER method). Similarly, in-hospital mortality was assessed by examining the occurrence of death during hospitalization relative to the full sample of admissions. Models controlled for the covariates listed previously. Thirty-day readmission was analyzed using the subsample of admissions of individuals who were discharged alive from their index admission, while in-hospital mortality was analyzed using the full sample of admissions. The referent group for comparing neighborhood median income was admissions from the lowest income quartile, for comparing insurance status was admissions from individuals on Medicare, and for comparing hospital location was admissions from large metropolitan hospitals. Admissions were clustered by facility; hence Generalized Estimating Equations (GEE) were applied to correct for standard errors. Odds ratios were calculated with 95% confidence intervals and p-values.

## Results

### Individual and hospital characteristics

A total of 13,986 unweighted admissions fitting the inclusion criteria were identified. Of those, 13,214 admissions were observed to be index hospitalizations and 693 were observed to be readmissions. The most commonly observed co-occurring physical and mental diagnoses were anxiety, depressive episode, and fluid, electrolyte and acid-base balance disorders. Further details on the summary statistics for the number of admissions and readmission rates for each characteristic are reported in Table 1.

**Table 1:** Baseline characteristics of admissions (*N* = 13,986) at index hospitalization and at 30-day readmission

Characteristic	Index admissions (*N* = 13214)	Readmissions (*N* = 693)	Index readmitted (%)
Payer			
Medicare	2036	164	8.06%
Medicaid	4200	202	4.81%
Private Insurance	6107	291	4.77%
Self-pay, no charge, and other	871	36	4.13%
Income Quartile			
1	2844	104	3.66%
2	3133	172	5.49%
3	3492	197	5.64%
4	3745	220	5.87%
Hospital Location			
Large metropolitan	8067	423	5.24%
Small metropolitan	4491	239	5.32%
Micropolitan and non-urban residual	656	31	4.73%
Age Group			
Children/ adolescents (8–17)	2581	99	3.84%
Young adults (18–30)	4512	247	5.47%
Midlife (31–55)	4820	278	5.77%
Older adults (56–64)	1301	69	5.30%
Sex			
Male	1885	76	4.03%
Female	11,329	617	5.45%
Length of Stay			
<4	4747	218	4.59%
4–7	4322	236	5.46%
>7	4145	239	5.77%
Discharge Location			
Routine	10,988	536	4.88%
Transfer to short-term hospital	176	12	6.82%
Transfer other	1246	85	6.82%
Home Health Care	711	57	8.02%
Co-occurring Diagnoses			
Anxiety	6948	392	5.64%
Depressive episode	6717	369	5.49%
Fluid, electrolyte, and acid-base balance disorders	3746	275	7.34%
Reaction to severe stress, and adjustment disorders	3126	184	5.89%
Malnutrition	3064	249	8.13%
Nicotine dependence	2850	159	5.58%
Gastro-esophageal reflux disease	2351	191	8.12%
Bipolar disorder	2062	127	6.16%
Hypertensive diseases	1960	104	5.31%
Volume depletion	1861	136	7.31%
Number of Co-occurring Illnesses			
0	798	21	2.63%
1–3	9083	393	4.33%
4–6	3240	267	8.24%
7–9	93	12	12.90%
Hospital Ownership			
Public	1780	94	5.28%
Private, non-profit	10,211	543	5.32%
Private, for-profit	1223	56	4.58%

### Independent predictors of readmission

The results of the multivariable logistic regression are displayed in Table 2. Lower odds of readmission were observed in admissions of Medicaid (OR = 0.71, 95% CI = 0.57–0.89), privately insured (OR = 0.66, 95% CI = 0.53–0.83), and self-pay individuals when compared to admissions of Medicare individuals (referent group). Separately, higher odds of readmission were observed among admissions of individuals in each income quartile when compared to the lowest income quartile (OR = 1.60, 95% CI = 1.23–2.10 in the highest income quartile when compared to the lowest income quartile). Odds of readmission did not significantly differ between hospitals located in different geographic areas, nor did they significantly differ between hospitals with varying ownership types. A statistically significant higher odds of readmission was observed among admissions of individuals co-diagnosed with malnutrition (OR = 1.49, 95% CI = 1.21–1.83), gastro-esophageal reflux disease (OR = 1.37, 95% CI = 1.12–1.67), or bipolar disorder (OR = 30, 95% CI = 10.3–1.64) compared to those without these diagnoses.

**Table 2:** Odds ratios and 95% confidence intervals of independent predictors associated with 30-day readmissions (*N* = 13,986)

Characteristic	OR (95% CI)	*P* Value
Payer		
Medicare	1 (Reference)	
Medicaid	**0.71 (0.57–0.89)**	**0.002**
Private Insurance	**0.66 (0.53–0.83)**	**0.000**
Self-pay, no charge, and other	**0.64 (0.44–0.92)**	**0.016**
Income Quartile		
1	1 (Reference)	
2	**1.46 (1.15–1.85)**	**0.002**
3	**1.51 (1.19–1.91)**	**0.001**
4	**1.60 (1.23–2.10)**	**0.001**
Hospital Location		
Large metropolitan	1 (Reference)	
Small metropolitan	1.11 (0.94–1.31)	0.232
Micropolitan and non-urban residual	1.06 (0.75–1.51)	0.746
Age Group		
Children/ adolescents (8–17)	1 (Reference)	
Young adults (18–30)	**1.35 (1.01–1.79)**	**0.041**
Midlife (31–55)	1.16 (0.88–1.53)	0.286
Older adults (56–64)	0.95 (0.66–1.35)	0.768
Sex		
Male	1 (Reference)	
Female	1.27 (0.96–1.66)	0.090
Length of Stay		
<4	1 (Reference)	
4–7	1.16 (0.95–1.41)	0.138
>7	1.08 (0.86–1.35)	0.501
Discharge Location		
Routine	1 (Reference)	
Transfer to short-term hospital	1.28 (0.70–2.34)	0.423
Transfer other	1.22 (0.96–1.56)	0.111
Home Health Care	1.20 (0.89–1.61)	0.231
Co-occurring Diagnoses		
Anxiety	0.97 (0.82–1.14)	0.707
Depressive episode	1.08 (0.89–1.31)	0.464
Fluid, electrolyte, and acid-base balance disorders	1.22 (0.98–1.51)	0.071
Reaction to severe stress, and adjustment disorders	1.02 (0.84–1.24)	0.842
Malnutrition	**1.49 (1.21–1.83)**	**0.000**
Nicotine dependence	0.91 (0.73–1.13)	0.400
Gastro-esophageal reflux disease	**1.37 (1.12–1.67)**	**0.002**
Bipolar disorder	**1.30 (1.03–1.64)**	**0.030**
Hypertensive diseases	0.89 (0.68–1.16)	0.388
Volume depletion	1.00 (0.77–1.30)	0.991
Number of Co-occurring Illnesses		
0	1 (Reference)	
1–3	1.22 (0.77–1.92)	0.397
4–6	1.67 (0.93–3.01)	0.086
7–9	2.16 (0.86–5.46)	0.103
Hospital Ownership		
Public	1 (Reference)	
Private, non-profit	0.92 (0.74–1.15)	0.473
Private, for-profit	0.83 (0.59–1.16)	0.281

Statistical modeling was conducted using logistic regression analysis. Covariates controlled for included age group, sex, length of stay, discharge location, number of co-occurring physical and mental diagnoses and the specific diagnoses present, and hospital ownership. Bold estimates indicate significance at the 5% level

### In-hospital mortality

The number of deaths that occurred out of the full sample of hospital admissions across the different income quartiles and insurance statuses, along with the results of the multivariable logistic regression are shown in Table 3. In-hospital mortality was a rare event, occurring in only a small percentage of admissions, and was lower among admissions of privately-insured individuals (0.39%, OR = 0.48, 95% CI = 0.27–0.84) compared to admissions of Medicare individuals (1.35%).

**Table 3:** Incidence of death and odds ratios of independent predictors associated with mortality (*N* = 13,986)

Characteristic	Total Admissions (*N* = 13986)	Total Died	Percentage Died	OR (95% CI)	*P* Value
Payer					
Medicare	2217	30	1.35%	1 (Reference)	
Medicaid	4424	38	0.86%	0.85 (0.51–1.41)	0.529
Private Insurance	6433	25	0.39%	**0.48 (0.27–0.84)**	**0.010**
Self-pay, no charge, and other	–	–	–	–	–
Income Quartile					
1	2960	30	1.01%	1 (Reference)	
2	3329	24	0.72%	0.71 (0.41–1.25)	0.233
3	3708	19	0.51%	0.57 (0.31–1.04)	0.068
4	3989	25	0.63%	0.91 (0.50–1.64)	0.747

Statistical modeling was conducted using logistic regression analysis. Covariates controlled for included sex, length of stay, number of co-occurring physical and mental diagnoses and the specific diagnoses present, and hospital ownership. Age group and discharge location were omitted due to collinearity. Bold estimates indicate significance at the 5% level. Cell sizes less than or equal to 10 have been omitted in order to protect privacy

## Discussion

The current study examined the relationship between socioeconomic status and two key outcomes—30-day hospital readmission and in-hospital mortality—among individuals hospitalized for eating disorders, finding that sociodemographic factors play a significant role in shaping these outcomes.

### 30-Day hospital readmission

The higher odds of readmission seen amongst admissions of Medicare individuals is not surprising, as those under the age of 65 years do not qualify for Medicare unless they have serious disabilities, whether due to severe eating disorders or other types of disabilities. The condition that qualifies these individuals for Medicare– whether it be long-term severe eating disorder, a comorbid psychiatric illness, or other conditions– may make them more vulnerable to all-cause readmission. Another factor may be access to post-hospital care, as Medicare insurance provides more consistent coverage and reimbursement compared to other forms of insurance.

Further analysis of Medicare admissions reveals a negative association between age and readmission rates (Table 4), with 13.69% of admissions in young adults being readmitted compared to 7.08% and 7.56% in midlife and older adults, respectively. Moreover, an examination of insurance status and discharge location (Table 5) suggests that a higher proportion of Medicare individuals are discharged to higher levels of care rather than home, relative to those with other types of insurance. While specific details on the nature of these facilities are unavailable, these findings further support the notion that Medicare admissions are more likely to involve higher-risk individuals.

**Table 4:** Associations between age and readmissions in medicare admissions (*N* = 2200)

Characteristic	Index Admissions (*N* = 2036)	Readmissions (*N* = 164)	Index Readmitted (%)
Age Group			
Children/ adolescents (8–17)	–	–	–
Young adults (18–30)	263	36	13.69%
Midlife (31–55)	1200	85	7.08%
Older adults (56–64)	569	43	7.56%

Cell sizes less than or equal to 10 have been omitted in order to protect privacy

**Table 5:** Associations between insurance status and discharge location (*N* = 13,986)

Discharge location	Medicare	Medicaid	Private insurance	Self-pay, no charge, and other
Routine	70%	84%	87%	88%
Transfer to short-term hospital	1%	2%	1%	1%
Transfer other	16%	9%	8%	7%
Home Health Care	13%	5%	4%	4%

Somewhat surprising were the similar odds of readmission observed between individuals with Medicaid and those with private insurance. This could be an indicator of similar access to care, though prior studies have demonstrated those on public insurance have less access to care [30, 31]. Instead, there could be unmeasured differences in the two populations, such as illness severity, that could be offsetting differences in access to or quality of care.

Individuals from higher income postal code areas were found to have higher odds of readmission, contrary to our initial hypothesis. Notably, admissions of individuals from the lowest income quartile had a 38% lower likelihood of readmission within 30 days following their initial hospitalization relative to those in the highest income quartile, inconsistent with previous literature [21]. Although studies on all-cause hospital readmission generally find lower area SES individuals to have higher 30-day readmission rates, [12–15] readmission within 30 days of discharge for individuals with eating disorders may not necessarily be an indicator of poor quality of hospital treatment, as stated by Di Vitantonio et al. [20] On the other hand, severe eating disorders, especially those requiring hospitalization, are likely to have a chronic relapsing natural history, and thus readmission may be an indicator of individuals accessing care and being recognized quickly when their status deteriorates. Again, unmeasured factors, such as disease severity could be contributing to differences in readmission rates. Without data on post-discharge care and outcomes, it remains unclear whether socioeconomic factors directly influence care quality or if other factors are driving these results.

Although both higher neighborhood median income and Medicare status increased odds of readmission, these two predictors are conceptually distinct. A negative association was observed between neighborhood median income and Medicare status, with the proportion of individuals with Medicare decreasing at higher income quartiles. In the lowest income quartile, 17.5% of admissions were covered by Medicare, while only 12.8% of admissions in the highest income quartile had Medicare coverage.

In terms of hospital location, it was initially hypothesized that admissions of individuals from rural locations were more likely than those in other locations to be readmitted within 30 days of their index hospitalization. It is recognized that there is a significant lack of access to specialty mental health care in rural areas of the United States, [32] with an estimated 65% of non-metropolitan counties having no psychiatrists [33] and over 60% of rural residents in the United States residing in regions recognized as lacking sufficient mental health providers by the U.S. Department of Health and Human Services [34]. Furthermore, the statistics used in this study were based on data from 2019, prior to the large-scale rollout of telehealth and tele-mental health services in response to the COVID-19 pandemic, which has since been reauthorized by U.S. legislation for continued use. Nevertheless, the observed data showed no significant differences in readmission rates by metropolitan (large or small) versus micropolitan and non-urban residual locations. Further research with a more balanced representation of rural and urban samples would provide a more comprehensive understanding of the factors influencing readmission rates in diverse healthcare settings.

### In-hospital mortality

Concerning in-hospital mortality, individuals with Medicare had significantly higher odds of death compared to admissions of privately insured individuals. This potentially reflects the greater severity of illness in the Medicare group, which may be a contributing factor to their qualification for Medicare in the first place. However, since the difference observed between the number of deaths in those with Medicare (*n* = 30) and those with private insurance (*n* = 25) was only a very small number of deaths (*n* = 5), the results may be due to chance. Although not statistically significant, those residing in the lowest neighborhood median income area had higher in-hospital mortality compared to those in other areas. Admissions of individuals from higher-income postal code areas showed lower in-hospital mortality compared to their lower-income postal code counterparts. While this pattern could reflect greater access to timely follow-up care when coupled with their higher 30-day readmission rates, the potential impact of disease severity on these outcomes should also be considered. Further investigation is needed to explore whether these differences are driven by access to care, disease severity, or other underlying factors.

### Clinical and public health implications

The findings of this study carry implications for improving treatment strategies for people with eating disorders. Median household income in the postal code of residence may serve as a proxy for structural disadvantage and healthcare access, which could influence patient trajectories during and after hospitalization. Previous literature have found low SES individuals to be more likely to face logistical barriers, such as long waiting times and inadequate transportation services for follow-up appointments, compared to higher income individuals [16, 35]. Socioeconomic disadvantage may also confer challenges related to poor health literacy [36, 37] or difficulties in adhering to self-care regimens [36, 38]. While it is clear that disparities exist, it remains unclear whether lower readmission rates in these admissions indicate improved post-discharge conditions, reflect a lack of close follow-up within the 30 days after discharge, or some other factor. Future research examining longer post-discharge periods and including data on outpatient and other forms of non-inpatient care could help clarify these dynamics.

Interestingly, discharge plan was not associated with the risk of readmission, highlighting a potential area for further investigation into the effectiveness of discharge planning for these individuals. However, the current study did not differentiate between the types of facilities admissions were transferred to, nor specify whether admissions were discharged with planned follow-ups. Further exploration of different discharge strategies and their impact on readmission rates could provide valuable insights into improving outcomes for people with eating disorders and reducing future hospitalizations.

### Limitations

The results of this study should be considered in light of several limitations. First, there are constraints related to cross-state readmissions stemming from limitations in the NRD. Admissions of people with eating disorders who were initially hospitalized in one state and subsequently readmitted to a hospital in a different state were not captured by the data. As a result, individuals residing in a different state than where they were hospitalized in at index admission were excluded from this study. The exclusion of individuals admitted to hospitals in a different state from their residence may have introduced a potential bias in the results of 30-day readmission, particularly when stratified by geographic location and SES. It is plausible that individuals, especially those from low-income rural areas, may have sought care in hospitals located in neighboring states with better infrastructure and more adequate resources to meet their needs, potentially leading to an underrepresentation of certain demographic groups in the study and affecting the generalizability of the findings.

In addition, while 30-day readmission is a standardized and widely used metric in health services research, this timeframe may not capture clinically relevant readmissions occurring beyond this period, particularly for individuals with chronic or relapsing conditions such as eating disorders. Because these disorders often persist for several years [39], with relapses occurring over months to years rather than weeks, focusing solely on 30-day readmissions may underestimate the true burden. Future research should examine longer-term readmissions to better reflect the clinical course of eating disorders.

Another limitation is that the cutoffs for neighborhood median income were predefined by HCUP, which restricted our ability to adjust them for a more comprehensive analysis. Similarly, due to the lack of information on household size in the data, neighborhood median income could not be adjusted for household size. Furthermore, the type of hospital in which individuals were hospitalized (psychiatric vs. non-psychiatric) was not specified in the dataset, further limiting the scope of our analysis.

This study further faced limitations in its capacity to fully characterize social risk factors and care access and utilization that likely impact readmission rates, including transportation, accessibility, social support, and health literacy. Consequently, distinguishing whether readmissions stemmed from acute hospital failure or from continuity of care failure was not possible. Further research is necessary to assess the interplay of social obstacles and their influence on hospital readmissions. A prospective cohort design may provide deeper insights into the relative contributions of acute hospital care and continuity of care in influencing readmission rates.

The absence of race and ethnic data posed a further limitation to this study. Different population subgroups are likely to exhibit varying proportions of distinct ethnic groups. Such ethnic and cultural variations in the different population subgroups may have contributed to the differences in the readmission rates between the different demographic groups. Similarly, the findings of this study must be interpreted in the context of the U.S. healthcare system, which may limit their generalizability to other countries with different healthcare structures, policies, and resource availability. Socioeconomic disparities observed in this study may reflect specific systemic features of the U.S. context. Future research examining how healthcare system differences influence admission and readmission patterns in eating disorders is necessary to determine the extent to which these findings are system-specific or reflect broader trends.

Additionally, the scope of covariates might not have comprehensively captured all relevant factors, which may have impacted the robustness of the analysis. Further research with a broader range of covariates may offer a more concrete picture of the complex interplay of variables influencing the outcomes assessed in this study. Although the study included proxies for disease severity, such as malnutrition, gastro-esophageal reflux disease, and Medicare status, these factors do not comprehensively capture the full extent of disease severity at diagnosis, which may significantly influence readmission and in-hospital mortality. This study additionally did not control for the duration of the disease at diagnosis, which, along with severity, may also significantly impact these rates. Individuals with more severe or prolonged illnesses may have higher readmission risks and poorer outcomes, which could affect the results of our analysis.

Lastly, this analysis treated eating disorders as a single category. It is important to acknowledge that various subtypes of eating disorders may manifest within distinct population groups, potentially resulting in variations in the independent predictors among these different classifications of eating disorders. Future studies that stratify the analysis by eating disorder subtype may help further our comprehension of the factors which contribute to an increased risk of readmission following initial hospital care.

## Conclusion

Eating disorders are a significant public health concern due to their widespread prevalence, severe health implications, and high mortality rates. Socioeconomic disparities in access to care and health outcomes persist, with lower SES often linked with poorer health outcomes across a range of medical conditions. However, evidence exploring how SES influences health outcomes specifically in eating disorders remains limited. This study examined the relationship between SES and short term 30-day readmission and in-hospital mortality among admissions of people with eating disorders. Our findings revealed the odds of readmission to be higher among admissions of individuals in the highest income quartile compared to those in the lowest quartile. Odds of readmission were additionally higher in individuals with Medicare compared to those with all other forms of insurance. In-hospital mortality was highest among admissions of Medicare individuals and lowest among admissions of privately insured individuals. Notably, neither the geographic location of the hospital nor hospital ownership were associated with risk of readmission.

By focusing on readmission and mortality, this study contributes to a deeper understanding of disparities in the trajectory of care for people with eating disorders. Future research should investigate the mechanisms through which insurance status, income, and care transitions influence recovery trajectories and explore strategies to ensure equitable access to high-quality, continuous care across all socioeconomic groups.

## Data Availability

The data used in this study is from the 2019 Healthcare Cost and Utilization Project Nationwide Readmissions Database (HCUP-NRD), which is available from the Agency for Healthcare Research and Quality (AHRQ) upon request: https://cdors.ahrq.gov/.

## Abbreviations

HCUP: Healthcare cost and utilization project
NRD: Nationwide readmissions database
SES: Socioeconomic status
